# The essential role of methylthioadenosine phosphorylase in prostate cancer

**DOI:** 10.18632/oncotarget.7486

**Published:** 2016-02-18

**Authors:** Gaia Bistulfi, Hayley C. Affronti, Barbara A. Foster, Ellen Karasik, Bryan Gillard, Carl Morrison, James Mohler, James G. Phillips, Dominic J. Smiraglia

**Affiliations:** ^1^ Department of Cancer Genetics, Roswell Park Cancer Institute, Buffalo, NY, USA; ^2^ Department of Molecular Pharmacology and Cancer Therapeutics, Roswell Park Cancer Institute, Buffalo, NY, USA; ^3^ Department of Pathology, Roswell Park Cancer Institute, Buffalo, NY, USA; ^4^ Department of Urology, Roswell Park Cancer Institute, Buffalo, NY, USA; ^5^ Department of Translational Hematology/Oncology Research, Cleveland Clinic Taussig Cancer Institute, Cleveland, OH, USA

**Keywords:** prostate cancer, methionine salvage pathway, methionine cycle, methylthioadenosine phosphorylase, polyamine metabolism

## Abstract

Prostatic epithelial cells secrete high levels of acetylated polyamines into the prostatic lumen. This distinctive characteristic places added strain on the connected pathways, which are forced to increase metabolite production to maintain pools. The methionine salvage pathway recycles the one-carbon unit lost to polyamine biosynthesis back to the methionine cycle, allowing for replenishment of SAM pools providing a mechanism to help mitigate metabolic stress associated with high flux through these pathways. The rate-limiting enzyme involved in this process is methylthioadenosine phosphorylase (MTAP), which, although commonly deleted in many cancers, is protected in prostate cancer. We report near universal retention of MTAP expression in a panel of human prostate cancer cell lines as well as patient samples. Upon metabolic perturbation, prostate cancer cell lines upregulate MTAP and this correlates with recovery of SAM levels. Furthermore, in a mouse model of prostate cancer we find that both normal prostate and diseased prostate maintain higher SAM levels than other tissues, even under increased metabolic stress. Finally, we show that knockdown of MTAP, both genetically and pharmacologically, blocks androgen sensitive prostate cancer growth *in vivo*. Our findings strongly suggest that the methionine salvage pathway is a major player in homeostatic regulation of metabolite pools in prostate cancer due to their high level of flux through the polyamine biosynthetic pathway. Therefore, this pathway, and specifically the MTAP enzyme, is an attractive therapeutic target for prostate cancer.

## INTRODUCTION

**Figure 1:**
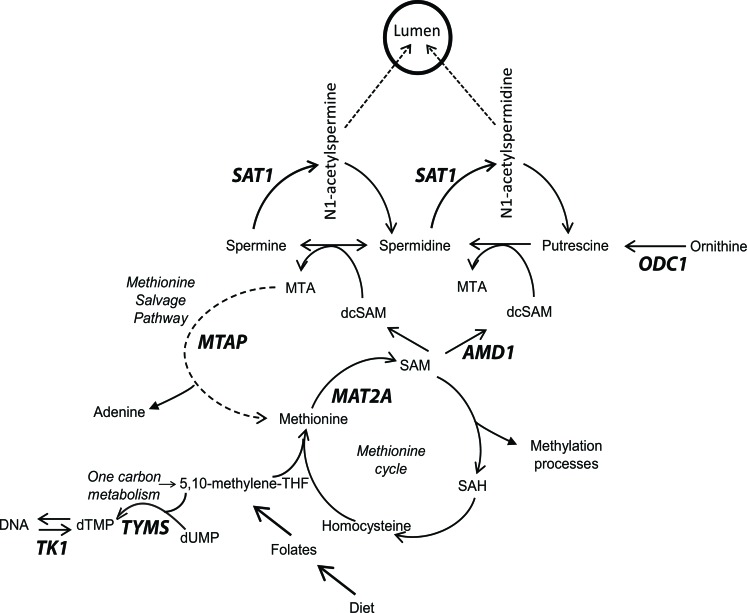
Overview of methionine cycle, polyamine biosynthesis and the methionine salvage pathway Key enzymes are bold. *MTAP*, Methylthioadenosine phosphorylase; *SAT1*, spermidine/spermine N1-acetyltransferase; *MAT2A*, methionine adenosyltransferase II alpha; *AMD1*, s-adenosylmethionine decarboxylase; *ODC1*, ornithine decarboxylase; *TYMS*, thymidylate synthase; *TK1*, thymidine kinase.

Prostate is a secretory gland that produces and secretes massive amounts of polyamines[1-4], ubiquitous molecules essential for cellular life. The secretion of acetylated polyamines is driven by the androgen regulated expression of spermidine/spermine N1-acetyltransferase (SAT1), which acetylates polyamines leading to their secretion into the lumen [5, 6]. This distinctive characteristic places added strain on connected pathways to maintain homeostatic control over intracellular polyamine pools when confronted with this export. Polyamine biosynthesis is connected to both the methionine cycle and one-carbon metabolism (Figure 1), which are therefore forced to increase metabolite production in order to maintain nucleotide and S-adenosylmethionine (SAM) pools, respectively [7-9]. This stress is enhanced in prostate cancer (CaP) due to increased polyamine biosynthesis, DNA synthesis, and proliferation [7-9]. The high flux through the polyamine biosynthetic pathway and connected pathways is enabled in part by the androgen driven expression of ornithine decarboxylase (ODC1) and s-adenosylmethionine decarboxylase (AMD1) [10, 11]. As a result, the prostate produces approximately 10 times more polyamines compared to other tissues [1-3].

SAM pools are consumed in order to synthesize polyamines by decarboxylation through the action of AMD1. This removes SAM and the one-carbon unit from the methionine cycle. The decarboxylated SAM donates the propylamine group to putrescine and spermidine through the actions of spermidine synthase (SRM) and spermine synthase (SMS), respectively. This results in the formation of spermidine and spermine, as well as the byproduct methylthioadenosine (MTA), which carries the one-carbon unit taken from the methionine cycle. Ultimately, this one-carbon unit is derived from one-carbon metabolism and requires folate derivatives to shuttle it to the methionine cycle for the synthesis of methionine and SAM. Therefore polyamine biosynthesis relies on folate from the diet *via* the generation of SAM.

We demonstrated that prostate cells require 5-10 times the amount of folate required by colon cancer cell lines [7, 9], where the effects of folate deficiency have been well studied [12-14]. This concept is further evidenced by the observation that the same mild dietary folate deficiency that was able to induce intestinal tumorigenesis in wild type mice within 12 months [15] and increase adenoma number in APC^min/+^ mice [16], instead led to arrest of CaP growth in 25 out of 26 TRAMP (Transgenic Adenoma of the Mouse Prostate) mice [8]. Escaping the growth suppressive effects of folate deprivation has been previously associated with upregulation of key enzymes involved in the synthesis and salvage of dTMP [8, 17]. Here we show for the first time that despite folate depletion, prostate maintains high SAM pools *in vivo*. In addition, folate depletion *in vitro* led to an initial decrease in SAM pools after 5, 10, and 15 population doublings (PDs) but they recover by PD 20. The recovery of SAM pools is associated with upregulation of methylthioadenosine phosphorylase (MTAP).

MTAP is the rate-limiting enzyme involved in the methionine salvage pathway (Figure 1), which recycles the one carbon unit lost to polyamine biosynthesis (contained within 5′-methylthioadenosine (MTA)) back into the methionine cycle in order to regenerate SAM [18]. This helps to relieve the stress placed on one-carbon metabolism and the methionine cycle by high rates of polyamine biosynthesis. Accordingly, MTAP is highly expressed in the prostate, from which it was originally purified [18-20]. The rat ventral prostate is known to secrete high levels of acetylated polyamines, and as a result has upregulated flux through the polyamine biosynthetic pathway and therefore produces high levels of MTA [21, 22]. Nevertheless, rat ventral prostate MTA levels are extremely low in comparison to other tissues, including the anterior prostate, which does not secrete high levels of acetylated polyamines [21, 22]. The high level of expression of MTAP in dorsal prostate is a likely explanation.

The MTAP locus is one of the most frequently deleted chromosomal regions in several types of human cancers due to its proximity to the CDKN2A (p16) gene on chromosome 9p21 [23-25]. However, examination of a variety of publically available datasets, cell lines, and tissue specimens indicates that loss of MTAP is rare in prostate cancer. Here we report that both normal prostate and prostate cancer uniquely protect SAM pools, even with depletion of folate levels, while maintaining a high level of polyamine biosynthesis. We suggest that MTAP plays a critical role in allowing this. Furthermore, we find that genetic and pharmacological based loss of MTAP function reduces prostate cancer cell line growth *in vitro*, and as xenografts. These findings strongly suggest that the methionine salvage pathway is a major player in homeostatic regulation of metabolic pools in prostate, and even more so CaP, making it an attractive therapeutic target.

## RESULTS

### SAM pools are protected in prostate cancer both *in vitro* and *in vivo*

**Figure 2:**
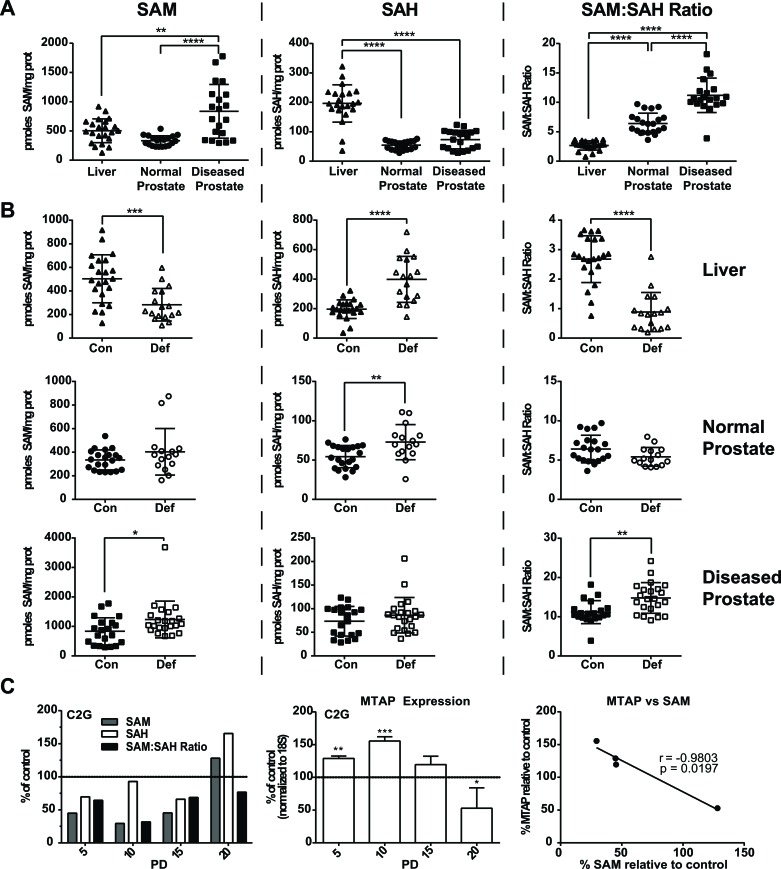
Analysis of SAM and SAH pools *in vivo* and *in vitro* **A.** SAM, SAH and SAM:SAHratios in the liver, normal and diseased prostate of TRAMP mice fed a folic acid control diet as measured by HPLC. Statistical analyses compare each of the three tissues to one another using a One-Way Anova with Tukey correction. **B.** SAM, SAH and SAM:SAH ratios in the liver, normal and diseased prostate of TRAMP mice fed a folic acid control (con) and deficient (def) diet. SAM:SAH ratios are decreased in the liver and increased in the diseased prostate of TRAMP mice fed a folic acid deficient diet. Statistical analyses were made using an unpaired student *t*-test. **C.** SAM and SAH measurements and MTAP expression by real time RT-PCR in TRAMP derived C2G cell line following folate depletion, relative to control. SAM pools were correlated to MTAP expression levels in the XY-scatter plot. Statistical analyses for MTAP expression compares folate control and deplete conditions using an unpaired student *t*-test; correlation calculated by 2-tailed Pearson correlation test (*: *p* < 0.05; **: *p* < 0.01; ***: *p* < 0.001;****: *p* < 0.0001).

Transgenic Adenocarcinoma of Mouse Prostate (TRAMP) mice were fed control folate defined diets from weaning until mice were sacrificed at 12 weeks of age as previously reported [8]. This model is driven by the prostate specific expression of SV40 large- and small- T-antigen upon puberty [26]. The anterior prostate has very limited expression of the transgene and is generally free of pathology at 12 weeks of age [26]. The dorsal, lateral and ventral lobes, however, highly express the transgene and by 12 weeks of age often exhibit high grade PIN or adenocarcinoma [26]. We used the anterior prostate from 12 week old mice as an approximation of normal prostate and took diseased tissue from the dorsal, lateral and ventral prostate. Analysis of SAM pools by HPLC in the liver, anterior prostate, and diseased prostate demonstrate that SAM levels are significantly higher in the diseased prostate when compared to the anterior prostate and liver (Figure 2a). Conversely, S-adenosylhomocysteine (SAH) levels are significantly lower in both the anterior and diseased prostate when compared to the liver, which results in a significantly higher SAM:SAH ratio (Figure 2a). Thus, in control conditions, the basal SAM to SAH ratios are ∼2.5 times and ∼4 times higher in the normal prostate and diseased prostate, respectively, than the liver.

TRAMP mice were also fed folate deficient diets from weaning until mice were sacrificed at 12 weeks of age as previously reported [8]. Prior studies have shown that folate deficiency leads to decreased SAM pools, as well as nucleotide pools [12-14]. Analysis of SAM pools in the liver of TRAMP mice demonstrates that dietary folate deficiency had a significant effect on SAM and SAH levels (Figure 2b). Specifically, folate deficiency induced a significant decrease in SAM pools and a significant increase in SAH, resulting in a significant decrease in the SAM:SAH ratio, as expected (Figure 2B - top panels). Conversely, we detected no change in the SAM:SAH ratio in the normal prostate (Figure 2B - middle panels). Strikingly, SAM levels in the diseased prostate were significantly higher in the deficient diet resulting in a significantly higher SAM to SAH ratio, opposite of what was predicted and seen in liver (Figure 2B - bottom panels). Notably, in mice fed a folate deficient diet, the SAM to SAH ratio is ∼6 times and ∼16.5 times higher in normal prostate and diseased prostate, respectively, than in the livers of the same mice. Importantly, we have previously reported that the folate deficient diet resulted in significant depletion of circulating serum folate as well as both liver and prostate tissue folates. Furthermore, despite successful depletion of tissue folates, prostate polyamine levels remain high [8]. These findings suggest the prostate has an adaptive mechanism that allows for compensation or adaptation to the strain placed on the system by dietary folate intervention.

We have previously reported that prostate cell lines grown for 20 population doublings (PDs) in folic acid (FA) deficient media (100nM FA) acquire an imbalance in the dUTP:dTTP ratio and that this is associated with increased DNA damage [9]. Similarly, we report that when TRAMP C2G prostate cancer cells are grown in 100nM FA, SAM and SAH pools are depleted compared to control as early as PD 5 (Figure 2C). This depletion is maintained through PDs 10 and 15, but rebounds at PD 20 (Figure 2C). This suggests the prostate has an inherent mechanism to protect SAM even under stressful conditions and we hypothesized that the methionine salvage pathway, controlled by the rate-limiting enzyme MTAP, may play an important role. Real time RT-PCR analysis of MTAP in TRAMP C2G cells showed that while SAM pools were depleted through PD15, MTAP expression was significantly upregulated compared to control (Figure 2C). Once SAM pools recovered by PD20 MTAP expression was significantly downregulated compared to the control (Figure 2C). SAM levels and MTAP expression levels were found to significantly and inversely correlate across population doublings 5-20 under folate restricted conditions (Figure 2C). These data suggest that MTAP is upregulated to compensate for the SAM pool depletion and may therefore be vital for maintaining growth in this metabolically strained environment.

### MTAP expression is retained in both CaP cell lines and CaP patients

**Figure 3:**
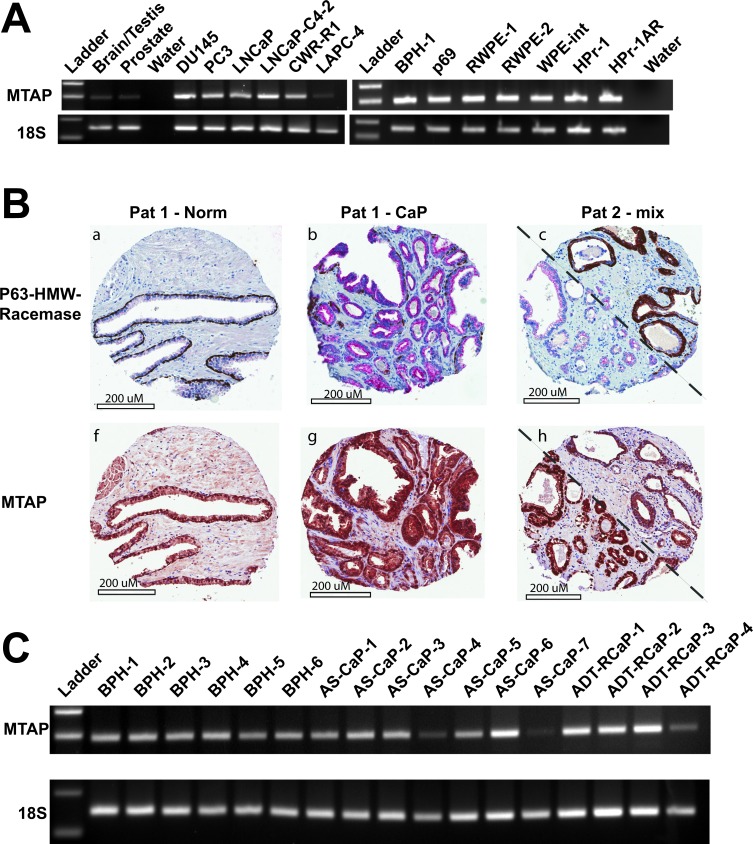
MTAP expression is conserved in normal prostate and prostate cancer **A.** Semiquantitative RT-PCR results (28 cycles) measuring 18S and MTAP in human brain/testis, human prostate, and 13 human prostate cell lines. **B.** Immunohistochemistry against P63-HMW-Racemase (top) and against MTAP (bottom) from a tissue microarray of 66 human prostate cancers and normal adjacent tissue. Representative cores are shown. In the top panels, brown staining indicates normal ducts or PIN; red staining indicates adenocarcinoma. MTAP staining is shown as brown staining in the bottom panels. Patient 1 has individual cores of normal (a and f) and cancer (b and g). Patient 2 (c and h) displays a mix of normal ducts/PIN and adenocarcinoma on either side of the diagonal. **C.** Semi-quantitative RT-PCR results (28 cycles) measuring 18S and MTAP in human benign prostatic hyperplasia (BPH), androgen sensitive CaP (AS-CaP), and androgen deprivation therapy recurrent CaP (ADT-RCaP).

If MTAP is required for prostate cancer to maintain growth, it would be expected that CaP would retain the MTAP locus despite its close proximity to the commonly deleted p16 locus. The TCGA provisional data set on CaP indicated homozygous deletion of MTAP in only 2 out of 498 cases. In the Memorial Sloan Kettering data set, MTAP deletion was seen in 3 out of 216 cases [27]. In the Stand Up to Cancer/Prostate Cancer Foundation Dream Team metastatic CaP dataset deletion was seen in 1 out of 150 cases. Overall, of the 9 publically available datasets, MTAP deletion was present in only 14 of 1543 total cases (www.cbioportal.org). This is in contrast to a number of studies in other cancer types that found deletion and/or mutation of MTAP at high frequency in: glioblastoma (GBM) 151/273 cases [28]; bladder urothelial carcinoma 39/127 cases [29], and in lung squamous cell carcinoma 46/178 cases [30]. mRNA expression by semi-quantitative RT-PCR (28 cycles) in a panel of 13 human prostate cell lines (both transformed and non-transformed) confirmed strong MTAP expression in all, regardless of androgen sensitivity or AR status (Figure 3A). Fluorescence *in situ* hybridization (FISH) using probes specific for either the MTAP or p16 loci on a human tissue microarray (TMA) comprised of prostate tissue from 75 patients indicated no cases of homozygous deletion at either locus (data not shown). Only 2 out of 75 patients exhibited a monoallelic deletion of the region of interest. MTAP expression analysis by immunohistochemistry on the same TMA confirmed that MTAP expression was detectable in 65 out of 66 cases (Figure 3B). No significant difference was observed in high or low MTAP staining comparing matched normal tissue to cancer. MTAP staining intensity and percent positive cells did not correlate with Gleason score.

To assess if MTAP expression was lost or changed during the process of CaP recurrence after androgen deprivation therapy (ADT), RNA was isolated from 6 samples of benign prostatic hyperplasia (BPH), 7 samples of androgen stimulated CaP (AS-CaP), and 4 samples of ADT-recurrent CaP (ADT-RCaP). MTAP expression was retained at all stages (Figure 3C) as shown by semi-quantitative RT-PCR (28 cycles). Real-time RT-PCR found no significant trend of increased or decreased expression from BPH, to AS-CaP, to ADT-RCaP (Supplemental Figure 2). These data demonstrate that the MTAP locus and expression are retained in CaP, regardless of cancer stage and/or patient androgen status, consistent with the idea that retention of MTAP might indeed be pivotal in sustaining CaP growth.

### MTAP contributes to CaP growth *in vitro* and *in vivo*

**Figure 4:**
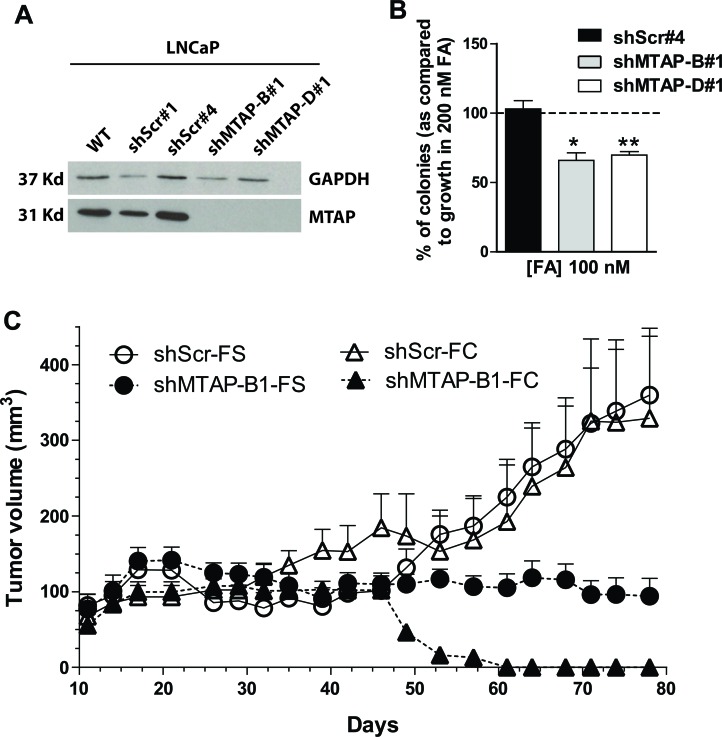
RNA interference of MTAP inhibits prostate cancer growth *in vitro* and *in vivo* **A.** Transfection with two shRNAs targeting MTAP (shMTAP-B#1 and D#1) in LNCaP cells decreases MTAP protein expression when compared to wild type or scramble control as measured by western blotting. **B.** Colony formation assay. Folic acid restricted conditions (100 nM) affects growth in LNCaP cells with MTAP knockdown or scrambled control shRNA compared to growth in 200 nM FA. Scrambled control cells are unaffected while both knockdown clones of MTAP show significantly reduced colony formation. *: *p* < 0.05; **: *p* < 0.01; *t*-test **C**. LNCaP xenograft growth in nude mice fed the folate control diet (FC - triangles) or a folate supplemented diet (FS - circles), with scrambled control (open symbols and solid lines) or MTAP shRNA (solid symbols and dashed line). 1×10^6^ cells in matrigel were injected into 20 nude mice for both control and MTAP knockdown lines. On the control diet and the supplemental diet, MTAP knockdown significantly reduces xenograft growth, *p* < 0.0001 and *p* = 0.013, respectively; *t*-test. Folate supplementation partially rescues xenograft growth in knockdowns.

To study MTAP contribution to CaP growth we used RNA interference to generate pure populations of LNCaP clones with stably silenced MTAP. We tested four shRNA sequences against MTAP (A-D) and found that both B and D were effective at significantly reducing MTAP protein (Figure 4A). Individual cells were isolated to create clonal populations of cells containing sequences B, D, and scrambled shRNA sequences predicted to target no proteins (shMTAP-B#1, shMTAP-D#1, shScr#1, and shScr#4). *In vitro*, LNCaP cells silenced for MTAP had growth rates comparable to control cells when grown in complete medium. Conversely, when grown under folic acid restricted conditions (100 nM folic acid), LNCaP cells silenced for MTAP, but not scrambled control cells, produced significantly fewer colonies when compared to the same cells grown in control medium (200 nM folic acid) (Figure 4B). These results indicate that MTAP partial loss of function caused by shRNA alone is insufficient to cause defects in proliferation *in vitro*, but instead requires additional metabolic stress such as restrictive levels of folate availability.

Subcutaneous injection of either 10^6^ LNCaP shScr#4 cells or 10^6^ LNCaP shMTAP-D#1 in nude mice (*n* = 20 per group) showed that indeed, MTAP knock down significantly (*p* < 0.0001) prevented the formation of tumors *in vivo* (Figure 4C). Dietary folate supplementation was able to partially rescue growth of LNCaP xenografts with MTAP knockdown (Figure 4C solid circles), suggesting that at least part of the xenograft growth inhibition caused by loss of MTAP was associated with downstream metabolic effects that could be partially mitigated by folate supplementation. Combined, these genetic data suggest that MTAP is required for CaP to grow *in vivo* and that MTAP loss of function impinges on one-carbon metabolism and the methionine cycle.

### Pharmacological inhibition of MTAP blocks growth *in vitro* and *in vivo*

**Figure 5:**
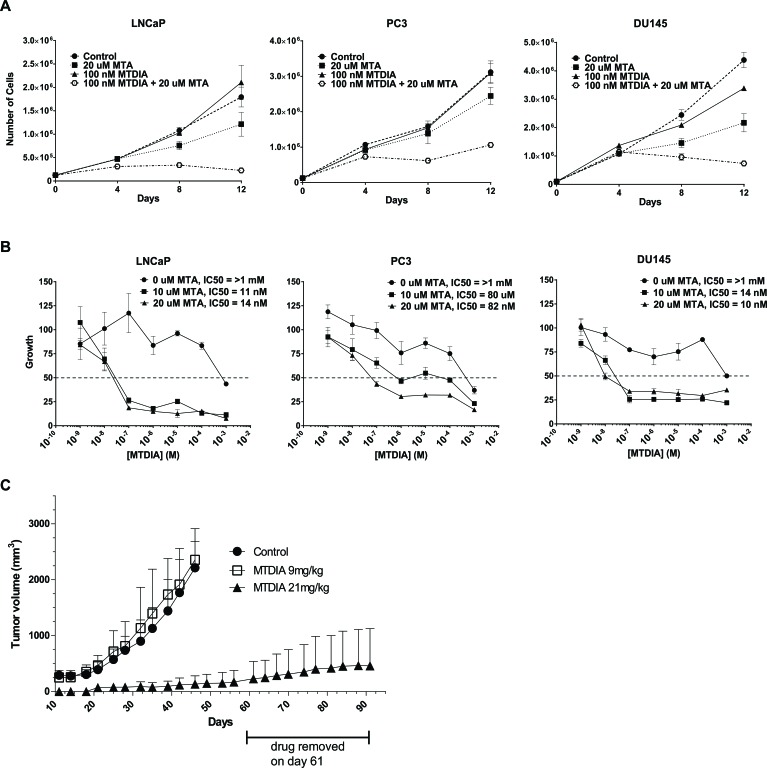
Pharmacological inhibition of MTAP blocks prostate cancer growth *in vitro* and *in vivo* **A.** Growth curves of 1 androgen sensitive (LNCaP), and 2 androgen insensitive cell lines (DU145 and PC3) treated with vehicle control, 100 nM MTDIA alone, 20 uM MTA, or 100 nM + 20 uM MTA for 12 days. 125, 000 cells/well in 6 well plates were plated, 24 hours later were refreshed with media containing 0, 10 or 20 uM MTA and treated with vehicle or 100 nM MTDIA, with drug and media replenished every 48 hours. Cells were trypsinized, counted by trypan blue exclusion and replated every 96 hours. Results of biological triplicates are shown. **B.** Proliferation curves after 12 days of treatment with MTDIA ranging in dose from 1nM to 1 mM, in the absence or presence of 10 uM or 20 uM MTA. Absolute IC50s are indicated for each condition. **C.** LNCaP xenograft growth in nude mice. 1×10^6^ wild type LNCaP cells in matrigel were injected into 60 nude mice. Twenty mice each were given the MTAP inhibitor MTDIA in the drinking water for a daily dose of either 9 mg/kg or 21mg/kg, and 20 control mice were given no drug. Drug was removed on day 61 for the 21mg/kg group, which was carried out to day 91. *p* < 0.001 *t*-test.

A transition state analog inhibitor of MTAP, Methylthio-DaDMe-Immucillin-A (MTDIA) was previously described and shown to be effective following a 6 or 12 day treatment at ∼10 and 100 nM MTDIA when 20 uM MTA was added to the media in both lung adenocarcinoma cell lines and head and neck squamous carcinoma cell lines, respectively [31, 32]. We synthesized MTDIA and the analytical data on the synthesized compound are shown in Supplemental Figure 3A and 3B with the scheme used for synthesis shown in Supplemental Figure 1. As shown in Figure 5A, 4 days of 100nM MTDIA treatment resulted in limited effects on growth for the LNCaP, PC3, and DU145 cell lines either with or without the addition of 20 μM MTA to the media. However, by 8 and 12 days there was a dramatic effect on cell growth for all three lines with the addition of 20 μM MTA, but little to no effect without it. In all three cell lines, the proliferation curves after 12 days of treatment show an IC50 of MTDIA > 1mM in the absence of MTA (Figure 5B), but in the 10-100 nM range with the addition of 10 or 20 μM MTA. Table 1 shows the IC50 values for all three cell lines at 4, 8, or 12 days with and without the addition of MTA to the media. Nanomolar range IC50 values are highlighted. IC50 curves at 4 and 8 days of treatment are shown in Supplementary Figure 4. These data demonstrate that MTDIA is effective for blocking prostate cancer cell lines with 8-12 days of treatment in the presence of MTA, similar to what has been previously shown in other models [31, 32].

**Table 1:** IC50 values for MTDIA in prostate cancer cell lines

	LNCaP			PC3			DU145		
	0 μM MTA	10 μM MTA	20 μM MTA	0 μM MTA	10 μM MTA	20 μM MTA	0 μM MTA	10 μM MTA	20 μM MTA
**4 days**	>1 mM	>1 mM	200 μM	>1 mM	>1 mM	300 μM	>1 mM	>1 mM	>1 mM
**8 days**	>1 mM	1 μM	**85 nM**	>1 mM	100 μM	**90 nM**	>1 mM	**22 nM**	590 μM
**12 days**	>1 mM	**11 nM**	**14 nM**	>1 mM	80 μM	**82 nM**	>1 mM	**14 nM**	**10 nM**

We subcutaneously injected 10^6^ wild-type LNCaP cells on the right flank of nude mice (*n* = 20 per group), and observed that treatment with 21 mg/kg MTDIA in the drinking water daily, caused a significant (*p* < 0.001) block in xenograft growth when compared to the untreated control and the 9 mg/kg dose (Figure 5C). In addition, upon drug removal at day 61 a significant block in xenograft growth was maintained (Figure 5C). These data show that pharmacological inhibition of MTAP is effective at blocking androgen sensitive CaP growth *in vitro* and *in vivo*.

Replicating previously published studies [31], we found the IC50 of MTDIA for FaDu cells in the presence of 20 uM MTA to be ∼50 nM following only a 6 day treatment (data not shown). However, for LNCaP, PC3 and DU145 cells 6 days had no effect at blocking cell growth at low concentrations of MTDIA (data not shown). For CaP cell lines 8 days is needed to see an effect of MTDIA. To further evaluate this difference we investigated molecular differences between the head and neck cell line, FaDu, compared to LNCaP and DU145. In the presence of 10 and 20 uM MTA for 4 days, MTAP protein expression is slightly increased in DU145, while in FaDu it is slightly decreased as measured by western blot (Supplemental Figure 4C). MTAP levels are unchanged in LNCaP upon MTA treatment. Strikingly, upon treatment with each cell line's respective IC25 and IC50 of MTDIA in the presence or absence of MTA, FaDu downregulates MTAP while both CaP cell lines upregulate expression of MTAP. In addition, spermine synthase is downregulated in FaDu cells upon treatment with MTDIA, but upregulated in LNCaP cell lines. These Western blot results were reproducible in biological triplicate experiments. This suggests that there are inherent mechanistic differences between the two types of cell lines in their ability to deal with accumulation of MTA, and that CaP cells can partially compensate for MTAP inhibition or increased MTA concentrations by upregulating both MTAP and spermine synthase in the initial stages of a 4 day treatment. Therefore, this could account for the cell line specific difference in length of time it takes to see MTDIA's effects. Nevertheless, MTDIA is highly effective and potent for blocking androgen-sensitive CaP growth *in vitro* following long-term treatments and *in vivo*.

## DISCUSSION

MTAP is the rate limiting enzyme involved in the methionine salvage pathway. The high degree of polyamine biosynthesis in prostate makes this salvage pathway critical because decarboxylation of SAM is necessary to provide the propylamine donor required for generating spermidine and spermine, which also generates MTA as a by-product (Figure 1). The salvage pathway recycles MTA, which carries the one-carbon unit lost from the methionine cycle to polyamine biosynthesis, back to the methionine cycle (Figure 1). MTA is a strong inhibitor of polyamine biosynthesis through end product inhibition of spermine and spermidine synthase (SMS and SRM, respectively). In the absence of MTAP, MTA can be released from the cell by passive diffusion, which avoids the inhibition of SMS and SRM, however wastes one-carbon units [33]. The activity of MTAP not only optimizes elimination of MTA, but also reclaims adenine and methionine. The methionine can then be used to replenish SAM pools through the action of Methionine Adenosyltransferase II, Alpha (MAT2A).

De novo synthesis of methionine depends on folate, which is acquired from the diet. Because of its characteristically high level of polyamine biosynthesis, prostate has a higher dependency on folate relative to other tissues [7], which is reflected in a higher flux of one-carbon units through one-carbon metabolism and the methionine cycle [8, 11]. Indeed we found that normal prostate and diseased prostate maintain a ∼2.5 times and ∼4 times higher basal SAM to SAH ratio than the liver (Figure 2a). Unexpectedly, upon folate deficiency in mice, normal prostate and diseased prostate maintain a ∼6 times and ∼16.5 times higher SAM to SAH ratio than the liver (Figure 2b). Interestingly, we found that the TRAMP C2G prostate cancer cell line exhibited a drop in SAM pools upon folate deficiency, but that by 20 PDs the SAM pools recovered and this correlated with increased expression of MTAP while SAM pools were low (Figure 2C). These data suggested that MTAP might be important for CaP to maintain SAM pools under conditions of metabolic stress.

MTAP is one of the genes most frequently deleted in some types of cancer, likely due to its proximity to the CDKN2A/p16 locus [34]. This suggests that losing p16 gives cancer cells a greater growth advantage than retaining MTAP. It may be the case that deletion of MTAP is less harmful in cell types with relatively low flux through polyamine biosynthesis, the methionine cycle, and one-carbon metabolism. This does not seem to be the case for CaP cells. Here we show that the MTAP gene is rarely deleted (14 of 1543 cases) across publically available datasets for CaP, as well as in a panel of CaP cell lines and patient samples. These data are consistent with reports indicating that p16 deletion is rare in CaP.

We hypothesized that given its apparent critical role in homeostatic control of SAM pools in prostate, MTAP is necessarily conserved in most prostate cancers, making it a potential therapeutic target. We show here for the first time that RNA interference of MTAP can significantly block CaP xenograft growth in nude mice. In addition, dietary folate supplementation was able to partially rescue this growth inhibition, suggesting that at least part of the antiproliferative effect is related to metabolic deficits that can be corrected by higher intake of folate. Pharmacological inhibition of MTAP with 21 mg/kg MTDIA in the drinking water also resulted in a significant block in CaP xenograft growth, making MTDIA a novel potential therapeutic agent to be applied to CaP.

Previous reports suggest that the cytotoxic effect seen in cells treated with MTDIA is due to the build-up of MTA which blocks polyamine synthase activity and ultimately leads to depletion of polyamines [31, 32]. In tissue culture conditions, excess MTA generated as a result of MTDIA treatment is passively diffused from the cell. *In vivo*, MTA accumulates not only in the target tissue, but also in surrounding tissues as a result of MTAP inhibition organism-wide. Therefore, MTA released from the prostate or target tissue is unable to dissipate. This is unlike *in vitro* conditions where MTA is diluted as it is released into the media. Consequently, in order to see the effects of MTDIA treatment *in vitro*, extracellular MTA must be added to the media. Hence, the IC50 in FaDu without MTA is > 1.0 mM but with MTA is ∼50 nM following a 6 day treatment [31, 32]. Similarly, the IC50 in three CaP cell lines is > 1.0 mM in the absence of MTA but with 20 (LNCaP and PC3) or 10 uM MTA (DU145) is < 100 nM following an 8 day MTDIA treatment. Western blot analysis of the cellular response to a 4 day treatment with MTA and MTDIA demonstrated that while FaDu cells decreased MTAP and SMS levels, the prostate cell lines did the opposite. These findings are consistent with CaP cell lines requirement for a greater length of time with our observation that CaP cell lines require a greater duration of treatment to achieve the IC50 of MTDIA, and suggests that prostate cells are able to initially compensate for MTA accumulation. Such adaptability is fitting with their uniquely high degree of MTA production consequent to their high flux through the polyamine biosynthetic pathway. It has previously been demonstrated that when MTA levels are high, normal prostate can upregulate MTAP by androgen driven expression [22], and thereby protect itself from MTA accumulation. This may also account for the slightly higher effective MTDIA treatment needed for LNCaP xenografts (21 mg/kg in drinking water), compared to FaDu which required only 9mg/kg MTDIA in the drinking water [31]. Notably, genetic knockdown of MTAP in LNCaP cells completely blocked xenograft growth despite the fact that MTAP was unaffected in the surrounding tissues of the mouse. In addition, folate supplementation was able to partially rescue growth, which suggests that CaP growth inhibition is not solely a result of MTA buildup. The degree to which growth inhibition is due to MTA accumulation, and therefore inhibition of polyamine synthesis, *versus* metabolite deficits such as SAM pools, is an open question for future studies.

By studying prostate specific metabolism we pinpointed at least two ideal targets for CaP therapy; prostate's characteristically high dependency on folate, and the requirement for the methionine salvage pathway through the activity of MTAP. Previous studies have explored the use of anti-folate therapies, specifically methotrexate, to treat castration recurrent, chemotherapy refractory CaP [35, 36]. In this clinical setting, anti-folate therapies were met with mixed results. Further studies are necessary to elucidate the potential to use treatments that target prostate specific metabolic requirements such as antifolate therapy and methionine salvage pathway inhibition at various stages of CaP progression.

## MATERIALS AND METHODS

### Cell lines and human tissue procurement

TRAMP-C2G cells are a clonal CaP cell line derived from a TRAMP prostate tumor [37] and were a kind gift of Dr. Barbara Foster (Roswell Park Cancer Institute (RPCI), Buffalo NY). The human prostate cancer cell lines WPE-int, DU145 and PC-3 were purchased from the American Tissue Type Collection (ATCC, Manassas CA). p-69 cells [38] were a kind gift of Dr. Irwin Gelman (RPCI). The human prostate cancer cell lines LNCaP, LNCaP C4-2, CWR-R1, and LAPC-4 were a kind gift of Dr. James Mohler (RPCI). The epithelial non-transformed CaP cell line RWPE-1 and the transformed version RWPE-2 were a kind gift of Dr. Moray Campbell (RPCI). The epithelial immortalized non-transformed cell line HPr-1 and the derived AR positive HPr-1AR were a kind gift of Dr. Eric Bolton (University of Illinois) [39]. The head and neck squamous cell carcinoma cell line FaDu was a kind gift of Dr. Mukund Seshadri (RPCI). The human prostate samples, androgen-stimulated benign prostate (AS-BP), androgen-stimulated primary prostate cancer (AS-CaP), and recurrent primary tumors (RCaP) were obtained as previously described [40].

### Mice and dietary intervention

All the mouse work was carried out at the Department of Laboratory Animal Research at RPCI, as previously described [8]. Briefly, male TRAMP (Transgenic Adenoma of Mouse Prostate) mice [26], heterozygous for the Pb-Tag transgene, ([C57BL/6J X FVB]_F1_ background), were bred in the RPCI Institute animal housing facility in accordance with an Institutional Animal Care and Use Committee-approved protocol. Mice were weaned at 3 weeks of age. At the time of weaning the mice were randomly assigned to two cohorts of at least 25 mice characterized by different folate concentrations in their diet as previously described [8].

### HPLC analyses

HPLC analyses were carried out as previously described [11, 41-43]. Standards for SAM and SAH were purchased from Sigma. All analyses were carried out on a reverse-phase Econosil (C_18_) column (5 μm particle size, 4.6×250 mm) (Fisher Scientific) with a C_18_ guard column assembled on the Waters 2796 Bioseparation module of the Biopolymer Facility, at RPCI (Buffalo, NY).

### Prostate TMA

The RPCI_PrCa7 tissue micro array (TMA) was prepared and analyzed at the RPCI Pathology core facility. Three 1-millimeter (mm) tissue cores from over 75 formalin-fixed paraffin embedded donor blocks of prostatic adenocarcinoma were precisely arrayed into a new recipient paraffin block. Specimens for controls consisted of multiple cores of normal tissue from 10 different organs including heart, colon, kidney, adrenal, ovary, myometrium, brain, thyroid, lung, and prostate. One slice was stained by immunohistochemistry (IHC) with p63 and the high molecular weight racemase, which are overexpressed in cancer tissues, to distinguish normal, prostatic intraepithelial neoplasia, and prostatic adenocarcinoma. Other IHC were carried out on other slices, including MTAP (Protein Tech group inc, cat# 11475-1-AP), as previously described [44].

### Fluorescence *in situ* hybridization (FISH)

FISH was carried out on the same TMA with PCR-validated probes from the RPCI BAC library collection hybridizing to either p16 (RP11-149I2) or MTAP (RP11-70L8). A commercially available spectrum green CEP9 probe (Vysis, Downers Grove, Ill.) was co-hybridized with the probe of interest (spectrum orange) for evaluation of copy number gain or loss. Cutoff values for the determination of each FISH were established by manually scoring 200 nuclei from forty 0.6-millimeter cores representing normal tissue from 10 different organs. Cutoff values were then established by calculation of the mean plus three times the standard deviation of the number of normal cells with a false-positive signal. For all FISH done in this study a total of at least 200 nuclei were manually scored for every case.

### RNA interference of MTAP

293T cells were transfected in a 6 well plate with MTAP shRNA constructs (A, B, C, and D) from a pGIPZ lentiviral shRNA library or pGIPZ scrambled shRNA non-silencing control, and packaging plasmids psPAX2 (contains GAG/POL/REV/TAT) and pMD2.G (contains ENV). Lentiviral containing supernatant from 293T cells was removed at 48 and 72 hours, filtered through .45uM PES filter, and used to infect LNCaP cells. Silencing was assessed and sequences B and D were deemed best. Populations B and D were then sub-cloned to obtain pure clonal populations.

### Western blotting

Whole cell extracts were prepared in RIPA buffer (150 mM NaCl, 1.0% NP-40, 0.5% deoxycholate, 0.1% SDS, 50 mM Tris-HCl pH 8.0, 5 mM EDTA and 0.5 mM PMSF) supplemented with 1x proteinase inhibitor. Protein concentrations were determined by Bradford Assay (Thermo Scientific, Cat #1856209). Samples were resolved on polyacrylamide gels and transferred onto PVDF membranes (Biorad, Cat #162-0177). Blots were incubated with primary antibodies overnight at 4° and with secondaries for 1.5 hours at room temperature. Signals were visualized using Pierce ECL western blotting substrate (Thermo Scientific, Cat #32209) and exposed to film. MTAP for Westerns [45] was purchased from proteintech™ (Cat #11475-1-AP. SRM and SMS were purchased from Abcam^®^ (Cat #ab111884 and ab156879). B-actin antibody for Westerns [46] was purchased from Sigma-Aldrich (Cat #A5441) and GAPDH [47] was from Santa Cruz Biotecnology Inc. (Cat #sc-25778). Films were scanned using the Biorad ChemiDoc XRS, and band intensities were calculated using Image Lab^TM^. Intensity values were normalized to B-actin loading control band intensities and made relative to control treatment conditions.

### Clonogenicity assays

3,000 LNCaP cells either silenced for MTAP or transfected with the control vector, were seeded in a 6 well plate in duplicate in methionine-free and folate-free RPMI medium supplemented with 10% dialyzed serum, 10^−9^ R1881, 24 μM methionine (the minimal amount required by prostate cells to grow *in vitro*), 2 μg/ml puromycin, and folate as indicated (100 nM as a slight deficiency or 200 nM as control). Cells were grown till visible colonies formed (about three weeks). Cells were fixed in 1% methanol, stained with 1% Giemsa, and the colonies were counted.

### Human cell line xenografts with RNA interference

Male Athymic Nude mice were castrated and pelleted with testosterone to bring their serum levels up to that of an adult human male and then randomly assigned to two groups. 1×10^6^ clonally derived LNCaP cells transfected with either shScrambled control or shMTAP-B were subcutaneously injected onto the flank of each mouse. Within each group of scramble control and shMTAP, there were two cohorts of 10 mice each. Mice were fed either folic acid control or supplemented diets (diet detail above) for a total of 4 cohorts, 10 mice per cohort. The experiment was repeated once to give a total of 20 mice per cohort. Tumor measurements were assessed and recorded twice a week.

### IC50

The MTAP inhibitor (3R,4S)-1-[(9-Deaza-adenin-9-yl)methyl]-3-hydroxy-4-(methylthiomethyl)-pyrrolidine, or MT-DADMe-Immucillin-A (MTDIA) was synthesized following the synthesis scheme shown in Supplemental Figure 1 and based on that previously described [48-50]. Cells were seeded at 125,000 cells/well (PC-3, DU145, LNCaP) in triplicate in 2 mLs of RPMI medium supplemented with 10% fetal bovine serum, and 1% penicillin streptomycin. After 24 hours the MTDIA and/or MTA was added at the indicated concentrations. Both medium and drug were refreshed every 48 hours. Every 96 hours the cells were trypsinized, ∼2% of cells were removed and counted by trypan blue exclusion and the rest were replated into a larger vessel.

### Human cell line xenografts with pharmacological treatment

1 × 10^6^ wild type LNCaP cells were subcutaneously injected onto the flank of pelleted nude mice. At time of injection animals were randomly assigned to one of the following three groups; control, 9 or 21 mg/kg MTDIA in the drinking water with 20 mice in each group. Tumor measurements were assessed and recorded twice a week. Animals were sacrificed once tumors reached 2 cm^3^ or at the end point of the experiment (91 days).

## SUPPLEMENTARY MATERIAL FIGURES


