# Supplementary material for: The essential role of methylthioadenosine phosphorylase in prostate cancer
Source: Oncotarget. 2016 Feb 18;7(12):14380–93. doi: 10.18632/oncotarget.7486 (PMC4924722; doi:10.18632/oncotarget.7486)
Supplement: Supplementary file 1 [file oncotarget-07-14380-s001.pdf]

# The essential role of methylthioadenosine phosphorylase in prostate cancer

**Supplementary Material**

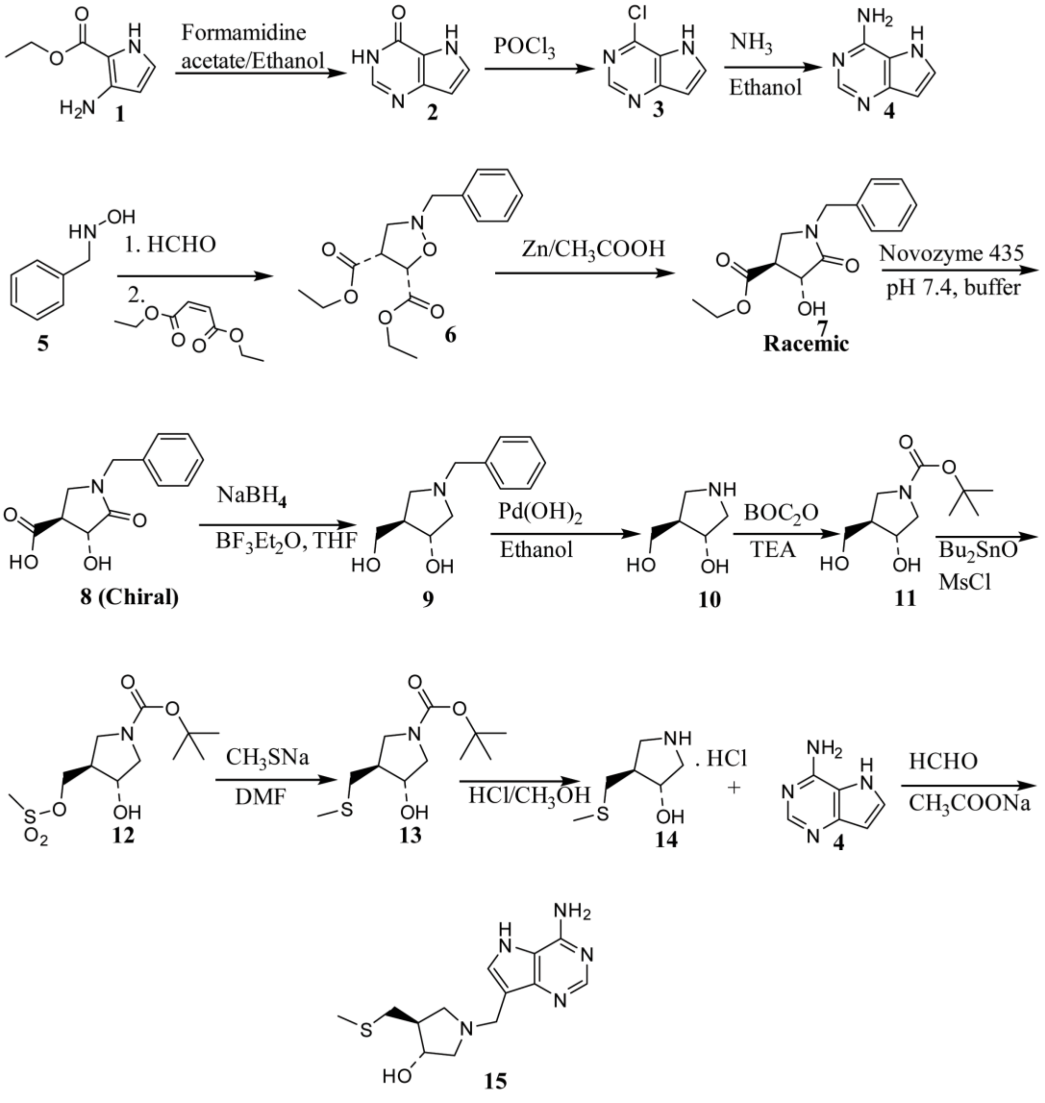


**Supplemental Figure 1:** Scheme I: Synthesis of (3R,4S)-1-[(9-Deaza-adenin-9-yl)methyl]-3-hydroxy-4-(methylthiomethyl)-pyrrolidine.

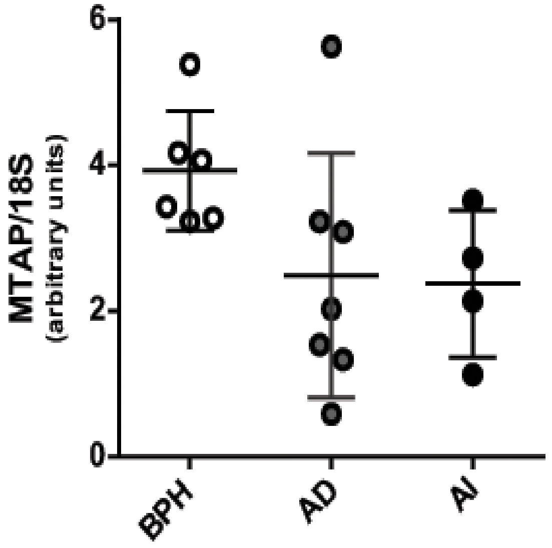


**Supplemental Figure 2:** MTAP expression in human prostate cancer.  No significant trend was seen in relation to stage of disease and MTAP expression in tissues representative of BPH, AS-CaP, and ADT-RCaP as measured by RT-PCR.

Data saved in:
chem400: /export/home/phillij5/vnmrsys/data

Archive directory: /export/home/phillij5/vnmrsys/data
Sample directory: phillij5_07Aug2015-13: 43: 19
File: PROTON

Pulse Sequence: s2pul

Solvent: DMSO
Temp. 25.0 C / 298.1 K
INOVA-400 "chem400"

Relax. delay 1.000 sec
Pulse 45.0 degrees
Acq. time 3.744 sec
Width 6395.9 Hz
8 repetitions
OBSERVE H1, 399.7395124 MHz
DATA PROCESSING

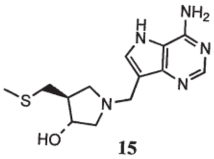


15

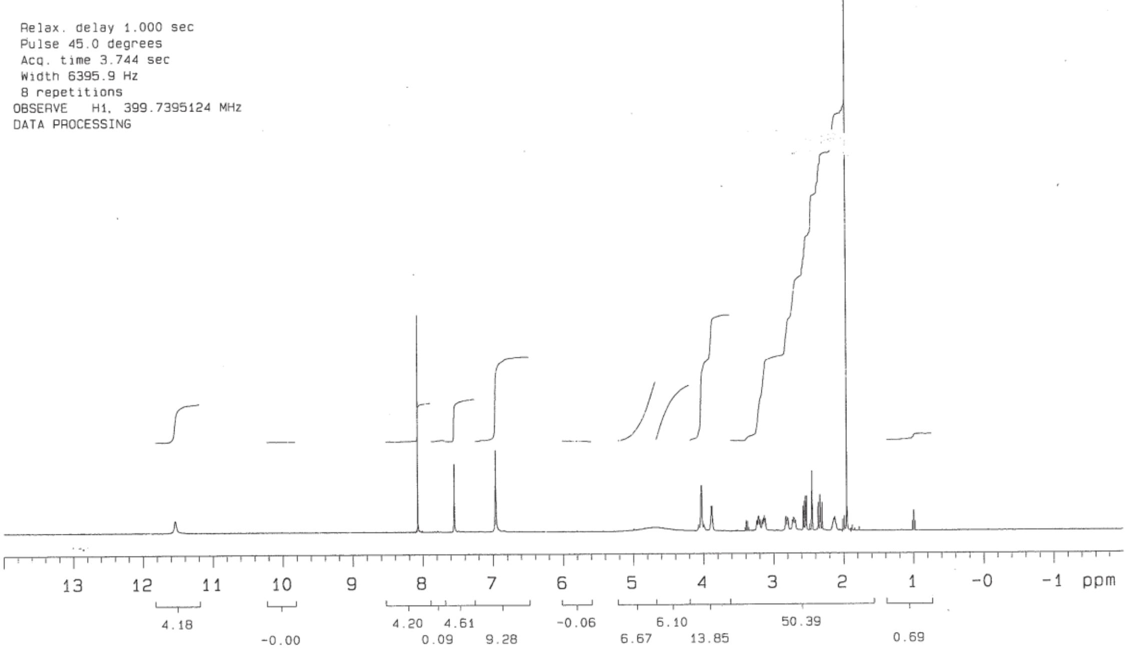

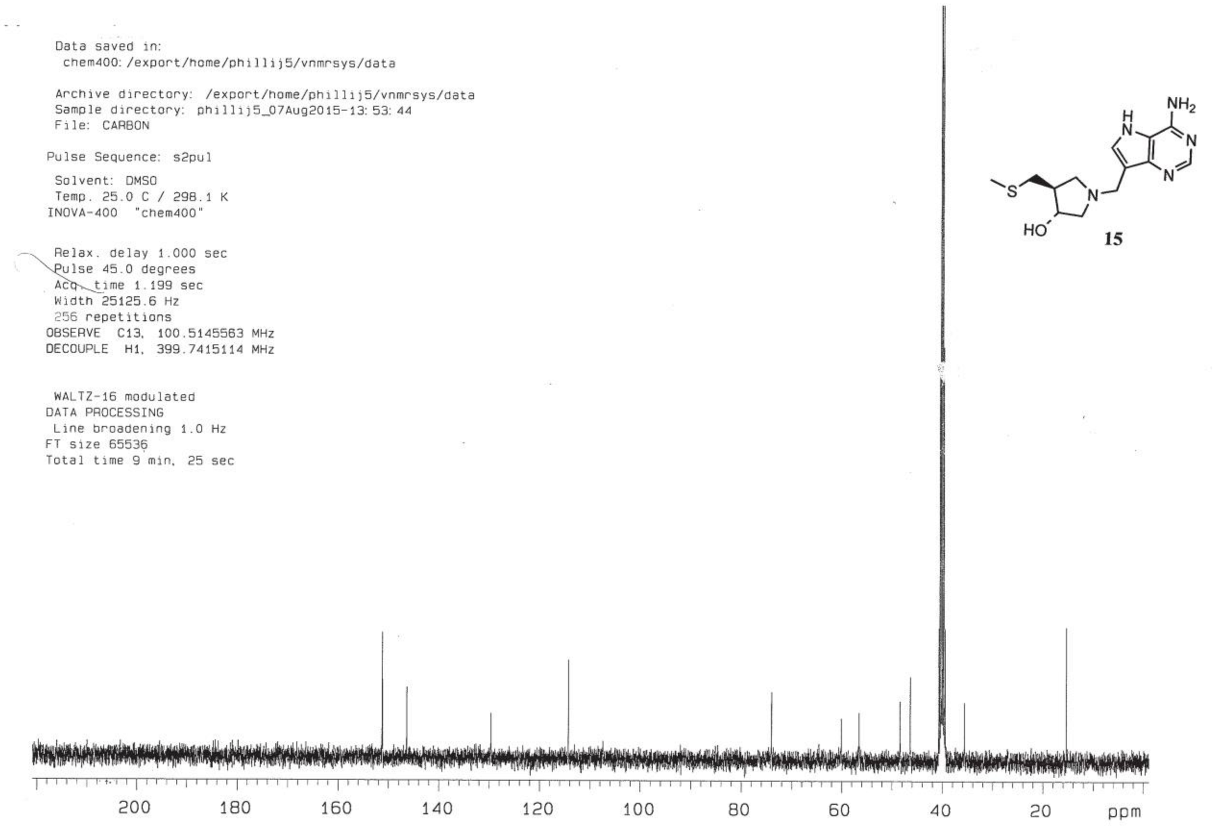


**Supplemental Figure 3:** Analytical data for (3R,4S)-1-[(9-Deaza-adenin-9-yl)methyl]-3-hydroxy-4-(methylthiomethyl)-pyrrolidine. A) $^{1}H$ NMR and B) $^{13}C$ NMR, and Mass Spectrum are consistent with structure 15 in the scheme shown in supplemental figure 1 and agree with similar data published previously (Compound 7 on page 4684 of J. Med. Chem., 2005, 48, 4679-4689) (38).

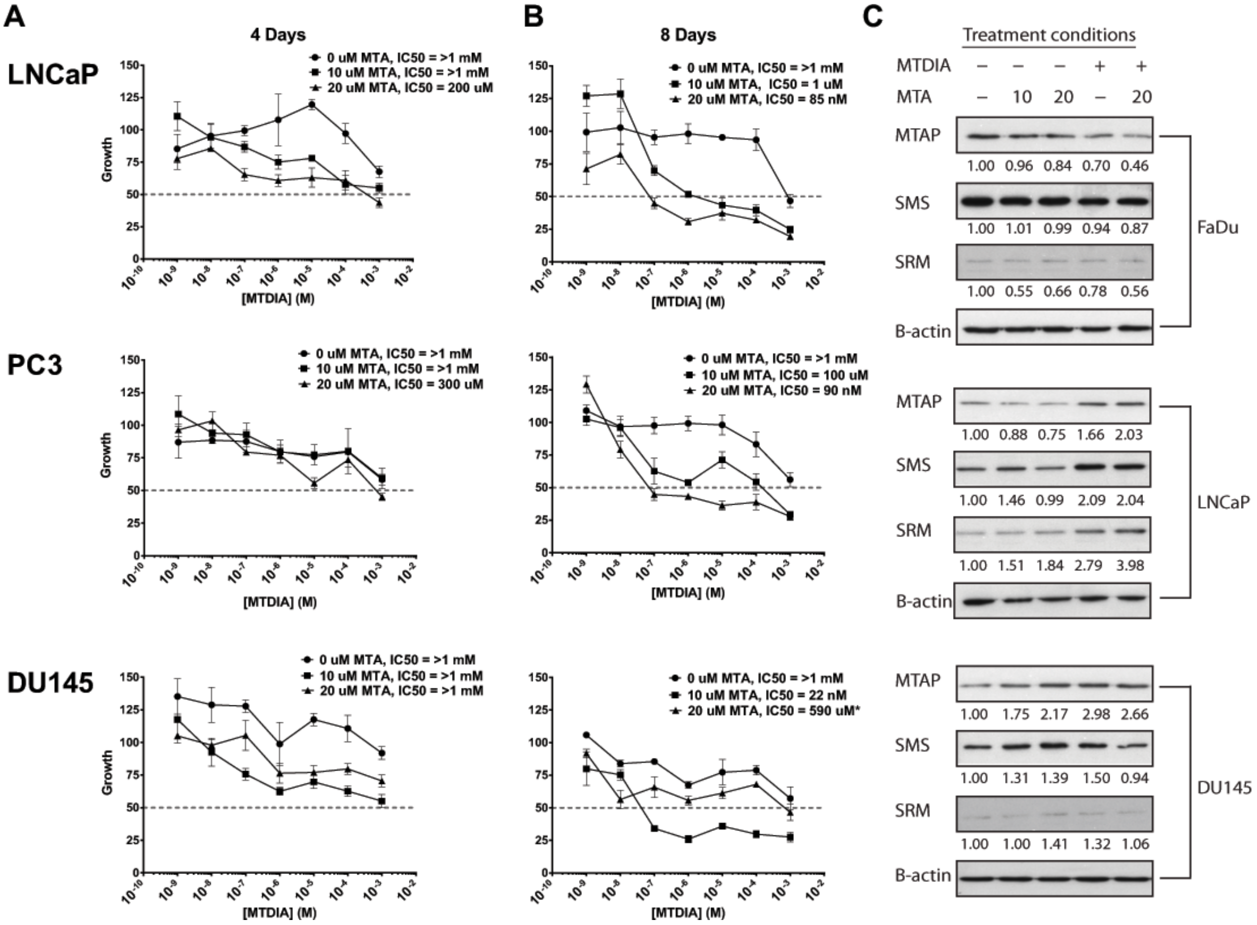


**Supplemental Figure 4:** (A) Proliferation curves for LNCaP, DU145 and PC3 treated with MTDIA in the absence or presence of 10 or 20 uM MTA, for 4 and (B) 8 days. Results of biological triplicates are shown with absolute IC50s indicated for each condition. (C) Western blot analysis of FaDu, LNCaP, and DU145 cells after indicated treatments of MTA (0, 10, or 20 uM) or the respective 4 day IC25 and IC50 dose of MTDIA for each cell line plus or minus MTA immunoblotted with antibodies against MTAP, SMS, and SRM, with B-actin as a loading control. Western blot images are representative of biological triplicates. Band intensity values normalized to B-actin relative to control conditions are indicated below the respective band.
